# The Effect of Polyhydroxylated Alkaloids on Maltase-Glucoamylase

**DOI:** 10.1371/journal.pone.0070841

**Published:** 2013-08-13

**Authors:** Qian Shang, Junfeng Xiang, Hong Zhang, Qian Li, Yalin Tang

**Affiliations:** Beijing National Laboratory for Molecular Sciences, Center for Molecular Sciences, State Key Laboratory for Structural Chemistry of Unstable and Stable Species, Institute of Chemistry, Chinese Academy of Sciences, Beijing, P. R. China; Department of Immunology, China

## Abstract

One of the most important carbohydrate-splitting enzymes is themaltase-glucoamylase which catalyzes the hydrolysis of alpha-glucosidic linkages. Maltase-glucoamylase inhibitors during the last few years have aroused medical interests in the treatment of diabetes. They contribute to a better understanding of the mechanism of maltase-glucoamylase. At present there are many different classes of maltase-glucoamylase inhibitors. This paper focuses on alkaloidal inhibitors of maltase-glucoamylase and structure-activity relationship (SAR) studies between them in order to discover some drugs with better efficiency and lower toxicity for treating diabetes.

## Introduction

Diabetes mellitus which is characterized by high blood sugar and metabolic disorder has been recognized as a severe health problem. Insulin-dependent diabetes mellitus (IDDM) (type 1 diabetes) [1] and non-insulin-dependent diabetes mellitus (NIDDM) (type 2 diabetes) [2], [3] are two different types of diabetes mellitus, but they share the same feature: the postprandial hyperglycemia (PPHG). The control of PPHG is needed in diabetes mellitus [4], [5], because PPHG has large damages by generating free radicals in the retina, renal glomerulus and so on [6]–[8].

Dietary disaccharides must be broken down into monosaccharides such as glucose and fructose in order to be absorbed by the small intestine. Maltase-glucoamylase (MGAM) which is one of membrane-bound enzymes in the small intestine can disassemble the disaccharides into monosaccharides. If its activity is inhibited, the digestion and absorption of monosaccharide can be slowed down, which is helpful for the decrease of PPHG. Thus the inhibitor of MGAM is significant for diabetes treatment. The MGAM inhibitor acarbose (Chart S1) which works by reversibly inhibiting MGAM [9], shows potential in reducing PPHG [10]–[12] in clinic. It is reported that MGAM inhibitors have made up a class of antihyperglycemic drugs as a way of controlling the postprandial glucose levels [13]–[16].

In China, there is a long history to treat diabetes mellitus with traditional Chinese medicines. Their potential in the treatment of NIDDM makes them attractive, such as *Coptis chinensis* [17], *Commelina communis* [18], *Corydalis* [19], *Anemarrhena asphodeloides* [20]. Such natural resources now become rich treasure [21] in the development of newer pharmacological agents for treatment of diabetes. It is reported that alkaloid is the main component in these traditional Chinese medicines [22]–[25]. After the first natural alkaloid nojirimycin (NJ) was extracted from *Streptomyces* in 1966 by Inouye etc.[26], there are 130 types of alkaloids isolated from plants and protozoa [27], [28]. In this work, alkaloids were screened from traditional Chinese medicines in order to explore their specific mechanisms against MGAM. In addition, structure-activity relationship (SAR) studies about alkaloids and MGAM were investigated in order to discover some drugs with better efficiency and lower toxicity for treating diabetes.

## Results and Discussion

### Molecular modeling of activity components in traditional medicines

Traditional medicines provide fertile ground for modern drug development. Here, some traditional medicines which are used for treating diabetes mellitus were focused. Among them, the alkaloid is one of the most important components (Chart S2). In this work, the alkaloids in these traditional medicines were chosen to investigate their interactions with N-terminal catalytic domain of MGAM (ntMGAM, PDB entry 2QMJ).

According to the crystal structure of the complex of acarbose/ntMGAM, the active site is defined as a pocket formed mainly by C-terminal β-strand residues of the (β/α)8 barrel structure [29]. In the center of the common grids, the center of mass coordinates of acarbose that had been removed from the binding site of the ntMGAM was used. The result was listed in Table 1. The dissociation constant of the complex of tetrahydropalmat/ntMGAM was smaller than that of the complex of acarbose/ntMGAM, which meaned the binding of tetrahydropalmat/ntMGAM was stronger than that of acarbose/ntMGAM. The tetrahydropalmat may be a potent ntMGAM inhibitor.

**Table 1:** Molecular docking study of main components from different plants and ntMGAM.

The complex	p*K*_d_
Tetrahydropalmat/ntMGAM	5.55
Palmatine/ntMGAM	4.80
Berberine_hydrochloride/ntMGAM	4.75
Harman/ntMGAM	3.98
Nicotinamide/ntMGAM	3.45
Acarbose/ntMGAM	4.92

p*K*_d_, the negative logarithm (base 10) of the dissociation constant.

### The characters of ntMGAM inhibitors

**Figure 1:**
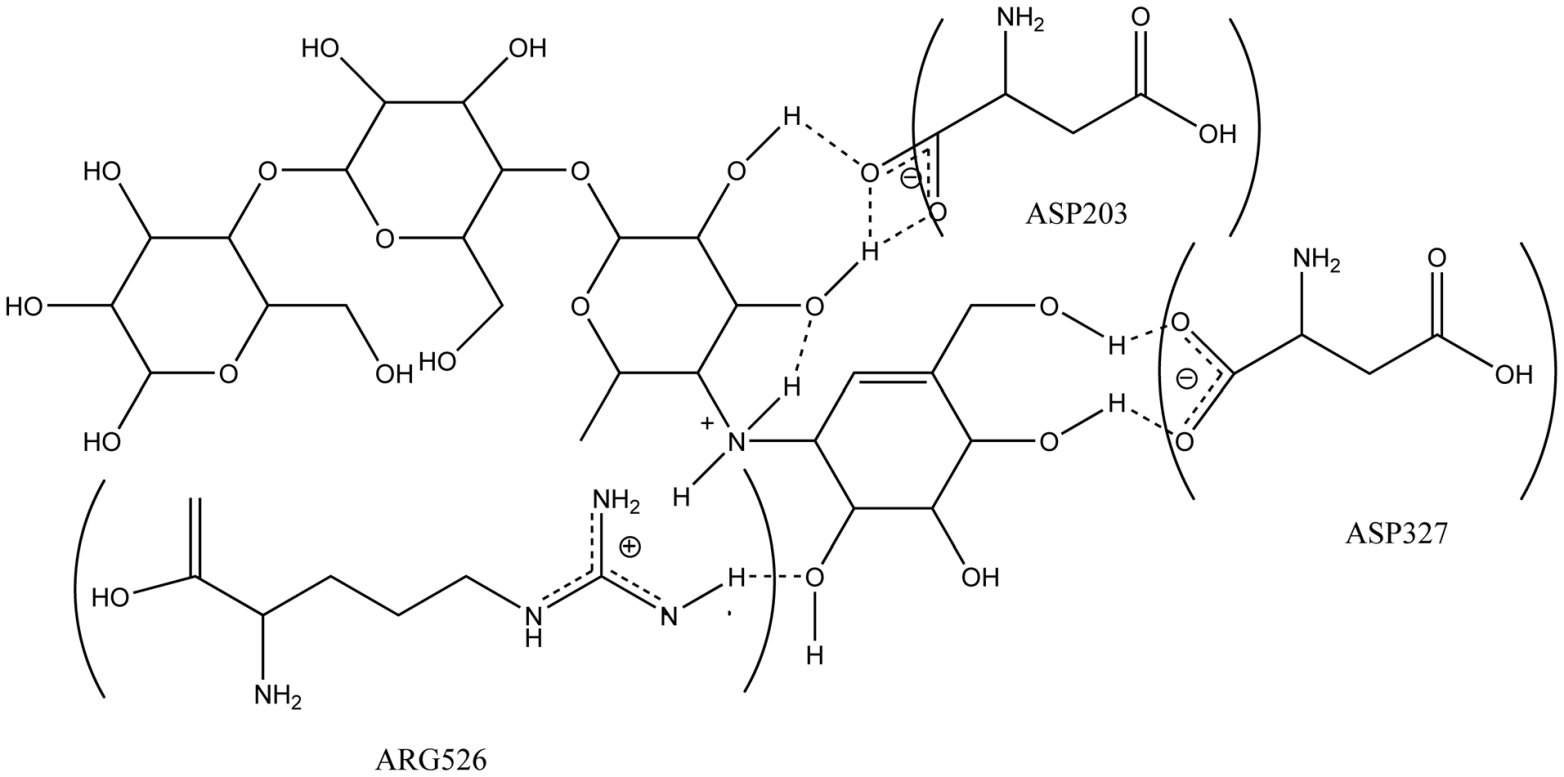
ntMGAM-acarbose interactions. Schematic representation of interactions (dotted lines) between the ntMGAM side chain residues and acarbose. (PDB entry 2QMJ).

The first character of ntMGAM inhibitors could be obtained from the interaction between acarbose and ntMGAM (Figure 1). Acarbose was tightly bound to ASP203, ASP327 and ARG562 of ntMGAM via hydrogen bonding in the active site [2]. It was inferred that compounds with hydroxyl groups were prone to interact with ntMGAM by hydrogen bonding.

Secondly, the nitrogen with positive charge which is benefit for approaching to ntMGAM contributed considerably to the ionic interaction between inhibitors and ntMGAM.

The hydroxyl groups and permanent positive charge are two important factors in the structure of ntMGAM inhibitors.

### The interactions between three new compounds and ntMGAM

According to the characters of ntMGAM inhibitors, three compounds (S1-b,S2-b and S3-b, Char S3) with nitrogen and different hydroxyl groups were designed and synthesized based on the structure of tetrahydropalmat which had the stronger binding to ntMGAM than acarbose according to above results.

**Figure 2:**
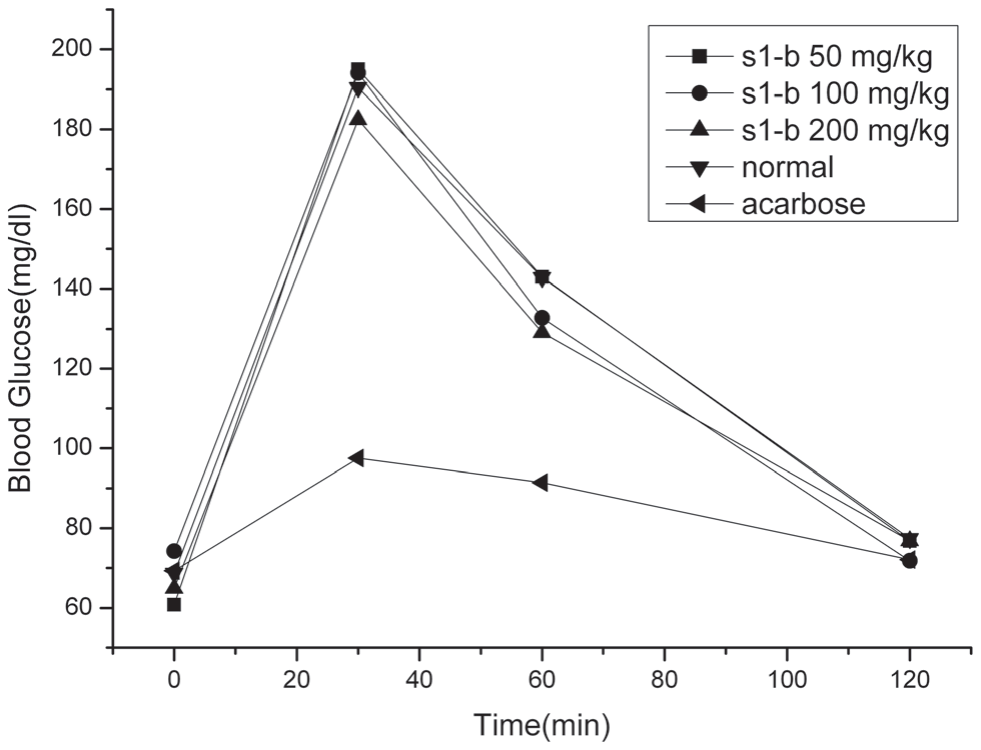
The influence of S1-b on blood glucose of normal ICR rat after sucrose loading. Acarbose (10 mg/kg of body weight); S1-b (50 mg/kg, 100 mg/kg, 200 mg/kg of body weight).

The molecular study was used to evaluate the interaction between three compounds and ntMGAM respectively. From the molecular docking study (Table 2), the p*K*d of the complex of S1-b/ntMGAM was highest in three compounds, which meant the bindings of S1-b/ntMGAM was strongest among three compounds. Then the influence of S1-b on blood glucose of normal ICR rat was present. (Table 3 and Figure 2) With the increase of concentration of S1-b, the blood glucose of normal rats decreased slightly. Further the interaction between S1-b and ntMGAM was studied.

**Table 2:** Molecular docking study of different compounds and ntMGAM.

The complex	p*K*_d_
S1-b/ntMGAM	6.48
S2-b/ntMGAM	6.16
S3-b/ntMGAM	6.21
Acarbose/ntMGAM	4.92

p*K*_d_, the negative logarithm (base 10) of the dissociation constant.

**Table 3:** The influence of S1-b and acarbose on blood glucose and area under curve (AUC) of normal ICR rat after sucrose loading (Student's t test).

Group	Dosage	Blood Glucose(mg/dl)				AUC
	(mg/kg)	0 min	30 min	60 min	120 min	(mg hr/dl)
Normal	0	68.7±2.6	190.4±15.9	142.8±31.4	77.4±10.6	258.2±25.9
S1-b	50	60.8±12.3	195.5±29.7	143.0±23.7	76.8±16.2	258.6±31.0
S1-b	100	74.3±8.2	194.1±34.2	132.7±24.3	71.8±5.5	251.0±12.3
S1-b	200	74.3±8.2	182.4±31.2	129.1±34.6	76.9±10.8	242.7±25.1
Aca	10	69.3±12.5	97.5±11.9***	91.4±9.4**	72.1±14.2	170.7±14.9***

Compared with normal,**P<0.01,***p<0.001;n = 8;X¯±SD. (dl = deciliter).

**Figure 3:**
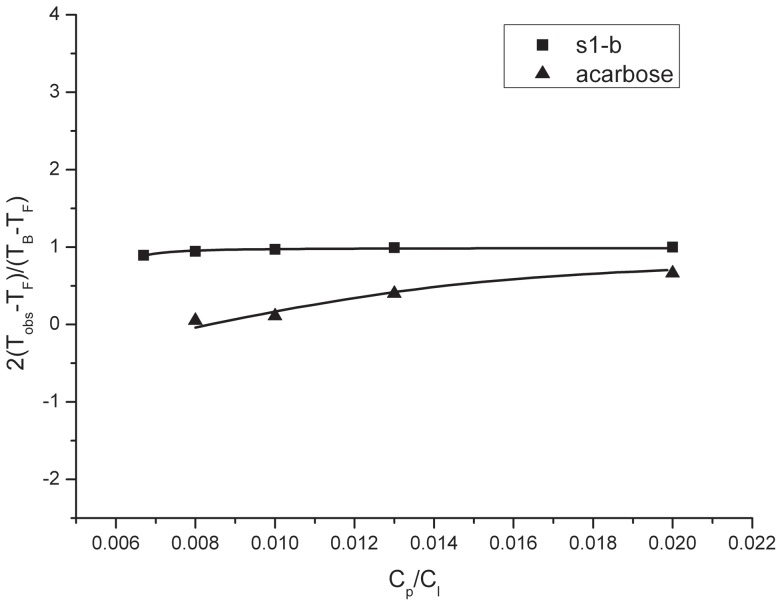
Plots of the measured relaxation time as a function of the concentration ratio *Cp/Cl* for the S1-b/ntMGAM system and acarbose/ntMGAM system. Solid curves are the calculated results using Eq. (2).

Nuclear magnetic resonance (NMR) was a powerful tool in study of ligand-target interaction [30]. For example, Luo etc.[31] investigated the drug-protein interaction by NMR diffusion and relaxation. The theory and equations used to obtain dissociation constant (*K*d) in this publication were suitable for obtaining the *K*d of inhibitor and ntMGAM. By fitting the data (Figure 3), *K*dS were obtained about the complexes of acarbose/ntMGAM and S1-b/ntMGAM respectively, as listed in Table 4. From this result, it was clear that the complex of S1-b/ntMGAM had the similar *K*d with that of acarbose/ntMGAM, which meant S1-b had the comparable binding capacity with acarbose to ntMGAM.

**Table 4:** Dissociation constant *K*_d_ for acarbose/ntMGAM system and S1-b/ntMGAM system complex determined by relaxation measurements.

δ^1^_H_(ppm)	*K*_d_(M^−1^)
Acarbose (H-9)	(5.4±0.1)×10^−^5
S1-b (H-11)	(1.3±0.4)×10^−5^

According to above experiments, it was proved that S1-b could interact with ntMGAM. S1-b with 200 mg/kg for normal ICR rats decreased the blood glucose of ICR rats slightly, which indicated the polyhydroxylated alkaloid could have the potential on decreasing blood glucose.

The structural analysis of the s1-b/ntMGAM complexes in this paper has also revealed interesting features relating to the binding mechanism of S1-b and ntMGAM (Figure S1). The electrostatic and hydrogen bond interactions between the ntMGAM side chain residues and S1-b were represented. Such information was advantageous to the design of the next generation inhibitors, which could help to discover the highly efficient and lowly toxic potential drugs.

Polyhydroxylated alkaloids are the main ingredient of the herbs with little toxicity and side-effects that have been administered to diabetic patients routinely. The knowledge gained from the analysis of the characters of ntMGAM inhibitors structures allowed us to explore variety of structural analogues of polyhydroxylated alkaloids in the search for more potent inhibitors of ntMGAM. In this work, polyhydroxylated alkaloids S1-b was designed and synthesized. Additionally, the NMR studies of S1-b/ntMGAM complexes supplied more information for rational design of inhibitors. This work highlighted the potential therapeutic use of polyhydroxylated alkaloids in the treatment of type-2 diabetes. We hope that such SAR studies will eventually provide us with lead candidates for the treatment of type-2 diabetes, which is the ultimate goal of our research.

## Conclusions

Alkaloid plays an important role in the traditional medicines used for treating diabetes mellitus. We compared the inhibition ability of three polyhydroxylated alkaloids (S1-b,S2-b,S3-b) and acarbose toward ntMGAM's activity and studied the structure-activity relationships about them. Additionally, S1-b is studied by molecular docking, vivo tests and NMR analysis. The result clews S1-b with higher concentration may have a potential to reduce blood glucose and polyhydroxylated alkaloids are promising compounds for diabetes mellitus treatment.

## Materials and Methods

### Ethics Statement

This study was performed in strict accordance with animal use protocols approvedby the standards for laboratory animals established by the People's Republic of China, laboratory animal requirements of environment and housing facilities (GB14925–2001). The use of all laboratory animals in this study was followed by the Beijing Laboratory Animal Welfare and Ethical Guidelines of the Beijing Administration Committee of Laboratory Animals (Permit Number: SCXK (jing)2002–2003). All animal experiments were approved by the Animal Ethics Committee of Chinese Academy of Medical Sciences. Mice were euthanized if they met any early removal criteria to limit suffering.

### Chemicals

Acarbose, maltase-glucoamylase from rice (Enzyme Commission (EC) Number 3.2.1.20, G9259) and glucose oxidase were purchased from Sigma-Aldrich Corporation. The other compounds were from Aladdin-Reagent Corporation.

### Synthesis of S1-b, S2-b and S3-b

S1-b, S2-b and S3-b were synthesized, and identified by mass spectrometry and NMR (shown in Figure S2, S3, S4, S5).

**S1-b:** BBr3 solution (1 M in CH2Cl2, 20 mL) was added to a stirred CH2Cl2 solution (30 mL) of l-tetrahydropalmatine (527 mg, 1.5 mmol). The reaction was stirred at −78°C for 25 min, and then was warmed to 0°C. The mixture was diluted with CH2Cl2 (180 mL) and washed with saturated NaHCO3, H2O and saturated NaCl. The organic extract was purified by silica gel column chromatography (CH2Cl2 andMeOH, 20∶1), followed by recrystallization in MeOH, to get S1-b.

**S2-b, S3-b:** BBr3 solution (1 M in CH2Cl2, 7.4 mL) was added to a stirred CH2Cl2 solution (30 mL) of l-tetrahydropalmatine (527 mg, 1.5 mmol). The reaction was stirred at −78°C for 25min, and then was warmed to 0°C. The mixture was diluted with CH2Cl2 (180 mL) and washed with saturated NaHCO3, H2O and saturated NaCl. The organic extract was purified by silica gel column chromatography (CH2Cl2 andMeOH, 20∶1), followed by recrystallization in MeOH, to get S2-b and S3-b.

### NMR experiment

The ntMGAM was dissolved in 0.01 M NaH2PO4-Na2HPO4 aqueous buffer at pH 6.8 and the concentration was 18 μM for all samples. 10% D2O was added in the samples to lock in NMR experiment. Bruker AVANCE 600 spectrometer equipped with a 5 mm BBI probe capable of delivering 50 G/cm z-field gradients was used in this work. The experimental temperature was at 310 K. Transverse relaxation times were measured by routinecpmg method, Δ = 1.5 ms, 2×n×Δ =  total spin-lock time and relaxation delays = 2 s. Water signal was suppressed using presaturation pulse. The association constant was calculated according to corresponding equation.

### Molecular modeling studies

AutoDock (v.4.0) software was applied in qualitative molecular modeling. The ntMGAM structure was obtained from the Protein Data Bank and the PDB IDs was 2QMJ [29]. Before the docking process, compounds and ntMGAM were optimized in a CHARMm force-field in Insight II 2005 (Accelrys, Inc.). The grid maps with default volume of 22.5×22.5×22.5 angstrom and a spacing of 0.375 angstrom were used. The maximum number of energy evaluations and Genetic Algorithm (GA) run were set to 2 500 000 and 100 during the docking procedure.

## Supporting Information

Figure S1 **ntMGAM-S1-b interactions.** Schematic representation of electrostatic and hydrogen bond interactions between the ntMGAM side chain residues and S1-b (PDB entry 2QMJ).
(JPG)

Figure S2 **The mass spectrum of the structure of S3-b.**
(PDF)

Figure S3 **The mass spectrum of the structure of S2-b.**
(PDF)

Figure S4 **The ^1^H-NMR of the structure of S3-b.**
(PDF)

Figure S5 **The ^1^H-NMR of the structure of S2-b.**
(PDF)

Chart S1 **The structure of acarbose.** Numbers show the location of carbons.
(TIF)

Chart S2 **The main components of active plants.**
(TIF)

Chart S3 **The structure of a) S1-b b) S2-b c) S3-b.** Numbers show the location of carbons.
(TIF)
