# Supplementary figures and images for: The Effect of Polyhydroxylated Alkaloids on Maltase-Glucoamylase
Source: PLoS One. 2013 Aug 13;8(8):e70841. doi: 10.1371/journal.pone.0070841 (PMC3742645; doi:10.1371/journal.pone.0070841)

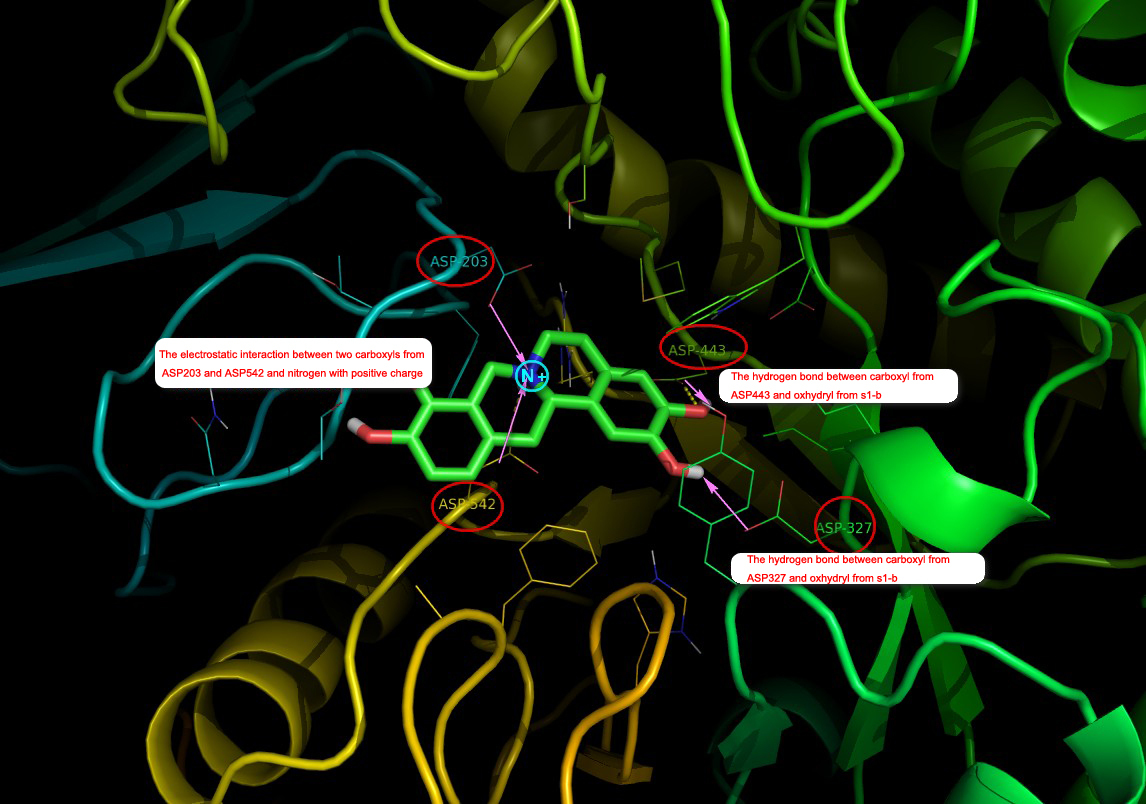

Supplement: Figure S1 — ntMGAM-S1-b interactions. Schematic representation of electrostatic and hydrogen bond interactions between the ntMGAM side chain residues and S1-b (PDB entry 2QMJ). (JPG) [file pone.0070841.s001.jpg]

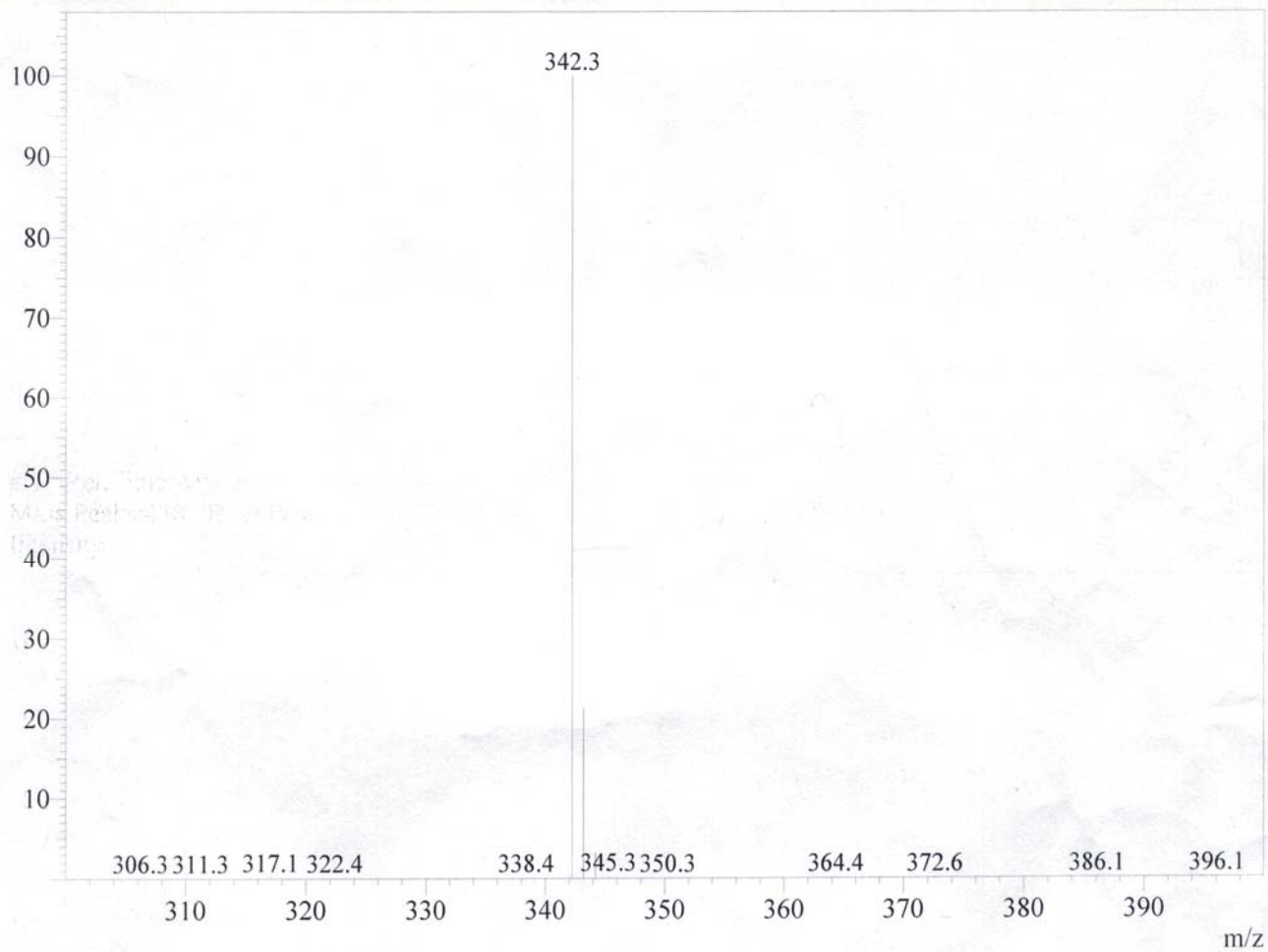

Supplement: Figure S2 — The mass spectrum of the structure of S3-b. (PDF) [file pone.0070841.s002.pdf]

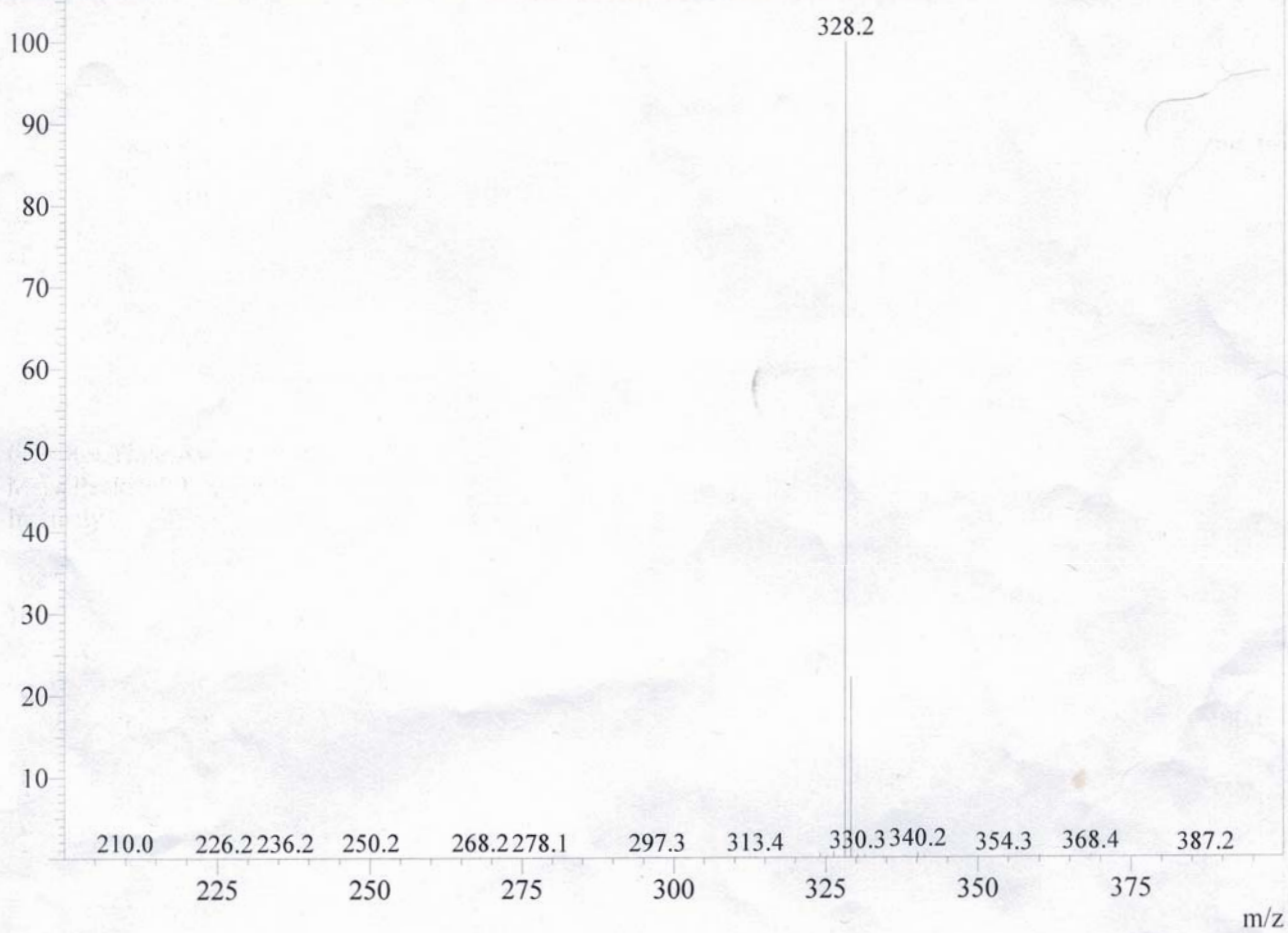

Supplement: Figure S3 — The mass spectrum of the structure of S2-b. (PDF) [file pone.0070841.s003.pdf]
