# Supplementary material for: The Effect of Polyhydroxylated Alkaloids on Maltase-Glucoamylase
Source: PLoS One. 2013 Aug 13;8(8):e70841. doi: 10.1371/journal.pone.0070841 (PMC3742645; doi:10.1371/journal.pone.0070841)
Supplement: Figure S4 — The 1H-NMR of the structure of S3-b. (PDF) [file pone.0070841.s004.pdf]

M1.

1H NMR
TET-5#
20110615

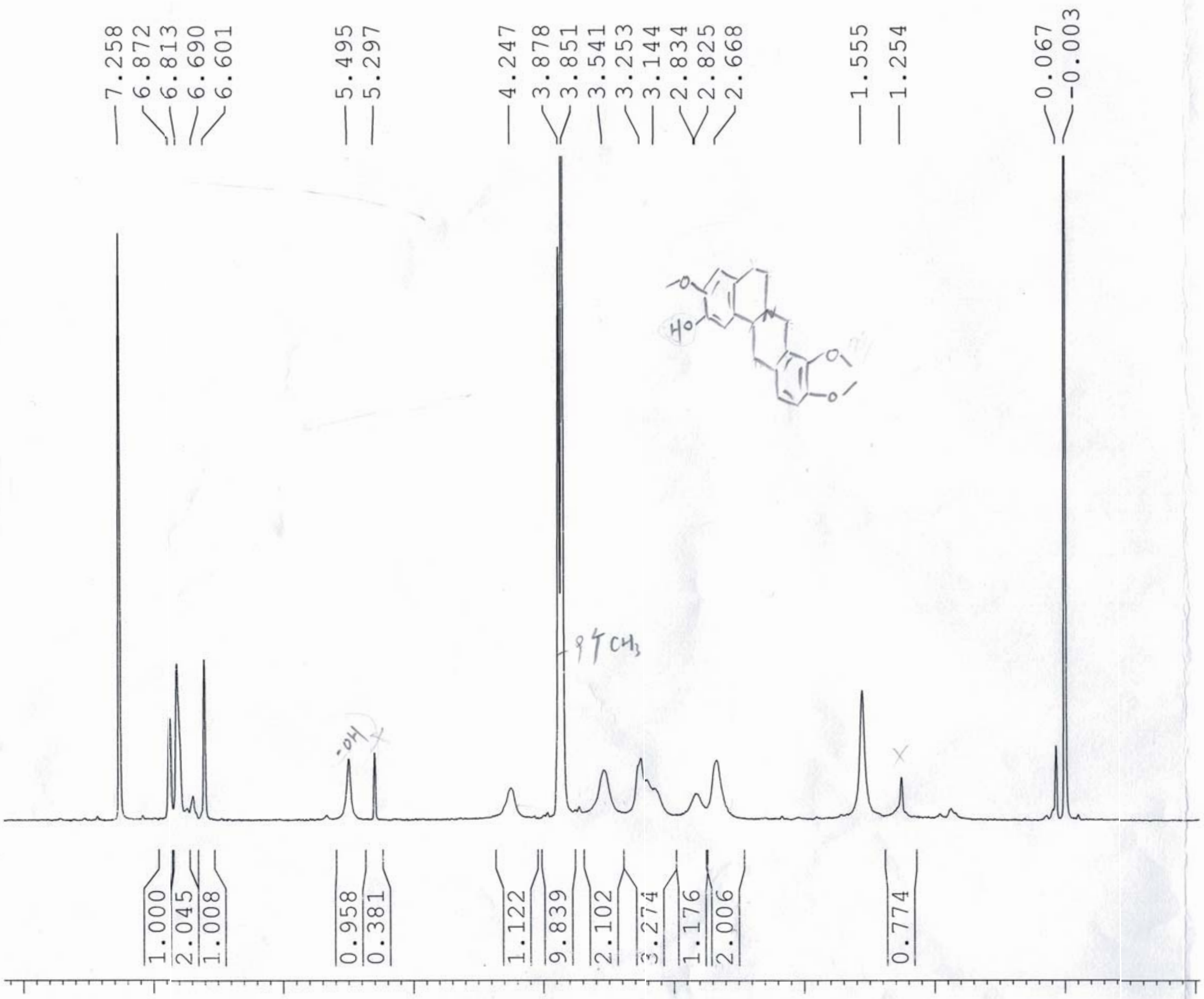

NAME	1h05
EXPNO	125
PROCNO	1
Date_	20110615
Time_	9.38
INSTRUM	spect
PROBHD	5 mm BBI 1H-BB
PULPROG	zg30
TD	32768
SOLVENT	CDCl3
NS	16
DS	0
SWH	17985.611 Hz
FIDRES	0.548877 Hz
AQ	0.9110282 sec
RG	812.7
DW	27.800 usec
DE	6.50 usec
TE	298.1 K
D1	2.00000000 sec
TD0	1

======== CHANNEL f1 ========

NUC1	1H
P1	11.80 usec
PL1	2.00 dB
PL1W	15.84893227 W
SFO1	600.1328788 MHz
SI	32768
SF	600.1300255 MHz
WDW	EM
SSB	0
LB	0.80 Hz
GB	0
PC	1.00
