# Supplementary material for: The Effect of Polyhydroxylated Alkaloids on Maltase-Glucoamylase
Source: PLoS One. 2013 Aug 13;8(8):e70841. doi: 10.1371/journal.pone.0070841 (PMC3742645; doi:10.1371/journal.pone.0070841)
Supplement: Figure S5 — The 1H-NMR of the structure of S2-b. (PDF) [file pone.0070841.s005.pdf]

m2

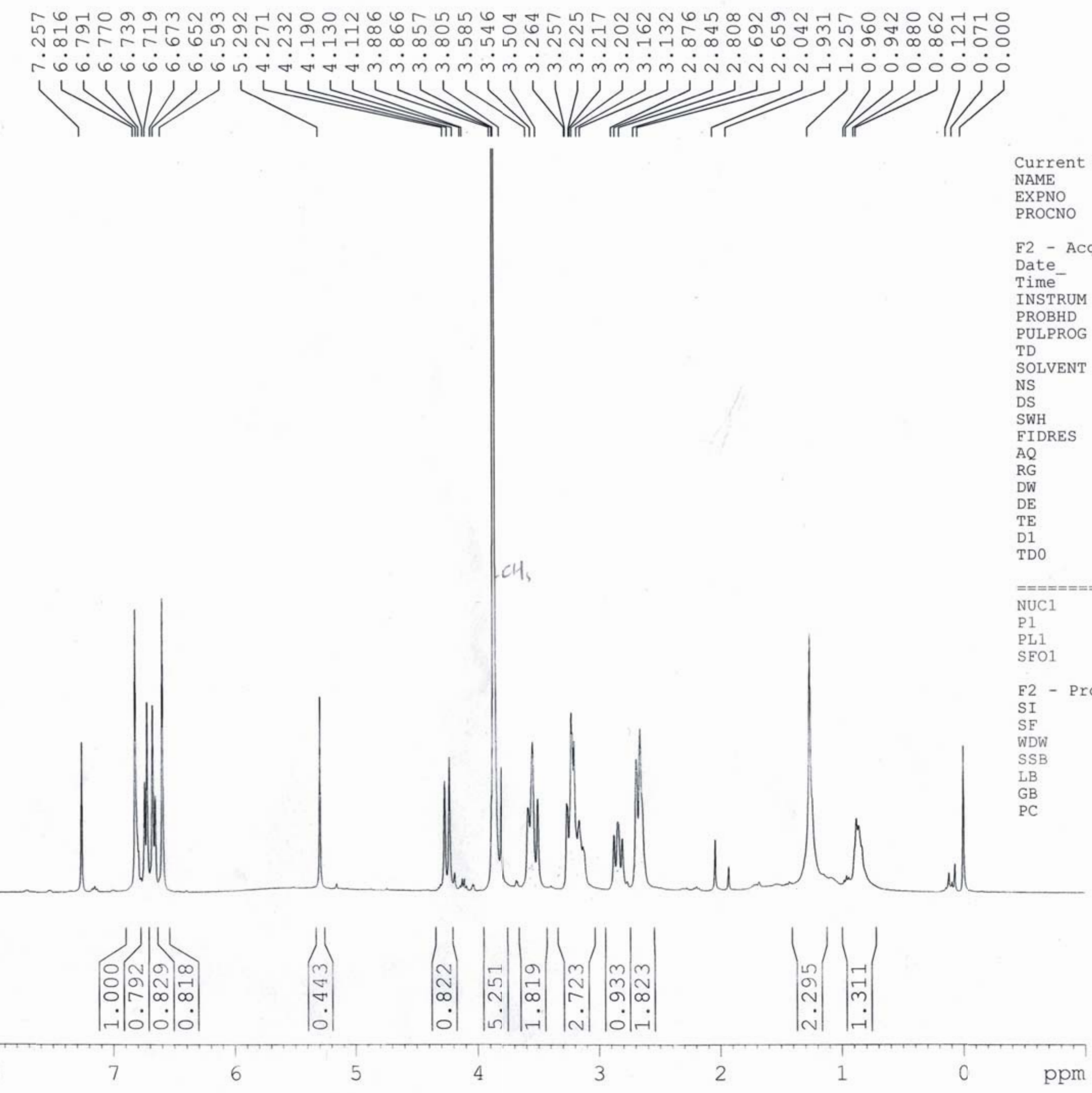

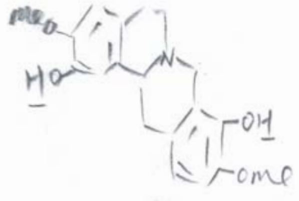

Current Data Parameters	
NAME	1h20110601
EXPNO	77
PROCNO	1
F2 - Acquisition Parameters	
Date_	20110617
Time	15.43
INSTRUM	spect
PROBHD	5 mm PADUL 13C
PULPROG	zg30
TD	32768
SOLVENT	CDCl3
NS	16
DS	0
SWH	12019.230 Hz
FIDRES	0.366798 Hz
AQ	1.3631988 sec
RG	203
DW	41.600 usec
DE	6.00 usec
TE	300.2 K
D1	2.00000000 sec
TD0	1
======== CHANNEL f1 ========	
NUC1	1H
P1	12.60 usec
PL1	-1.00 dB
SFO1	400.1320007 MHz
F2 - Processing parameters	
SI	32768
SF	400.1300126 MHz
WDW	EM
SSB	0
LB	0.80 Hz
GB	0
PC	1.00
